# The Association of Hypovitaminosis D with the Metabolic Syndrome Is Independent of the Degree of Obesity

**DOI:** 10.5402/2012/691803

**Published:** 2012-10-24

**Authors:** Inka Miñambres, Joan Sánchez-Hernández, Jose Luis Sánchez-Quesada, Jose Rodríguez, Alberto de Leiva, Antonio Pérez

**Affiliations:** ^1^Endocrinology and Nutrition Department, Hospital de la Santa Creu i Sant Pau, Sant Antoni Maria Claret 167, 08025 Barcelona, Spain; ^2^Medicine Department, Universitat Autònoma de Barcelona (UAB), 08193 Bellaterra, Spain; ^3^Diabetes and Metabolic Diaseases CIBER, CIBERDEM, Sant Antoni maria Claret 167, 08025 Barcelona, Spain; ^4^Biomedical Research Institute (IIB Sant Pau), Sant Antoni Maria Claret 167, 08025 Barcelona, Spain; ^5^Clinical Biochemistry Department, Hospital de la Santa Creu i Sant Pau, Universitat Autònoma de Barcelona (UAB), Sant Antoni Maria Claret 167, 08025 Barcelona, Spain; ^6^Bioengineering, Biomaterials and Nanomedicine CIBER, CIBERBBN, Sant Antoni Maria Claret 167, 08025 Barcelona, Spain

## Abstract

*Background*. It remains uncertain whether the metabolic syndrome (MS) or insulin resistance contribute to the association between vitamin D deficiency and obesity. *Methods*. We conducted a cross-sectional survey of 343 subjects who were overweight or obese. We analyzed anthropometric data and the presence or absence of MS. Additionally, we determined 25-hydroxyvitamin D (25OHD) and insulin concentrations, and the HOMA index was calculated. Chi-square test,Mann-Whitney *U* test, Student's *t*-tests,and logistic regression analysis were used. *Results*. The mean age of the patients was 42 ± 11 years, and 65.9% were women. The mean BMI was 34.7 ± 8.3 kg/m^2^ and 25(OH)D levels were 53.7 ± 29.8 nmol/L. Forty-six patients (13.4%) had MS. Vitamin D status was associated with the degree of obesity, especially with a BMI > 40 kg/m^2^. Patients with MS had lower levels of 25(OH)D than patients without (43.3 ± 29.0 versus 55.3 ± 29.6 mmol/L, resp.), and the odds ratio for hypovitaminosis D was 2.7 (confidence interval (CI), 1.14–6.4) (*P* = .023) for patients with MS versus patients without MS, irrespective of the degree of obesity. *Conclusions*. Our data confirm the association between vitamin D and MS and suggest that this association is independent of the degree of obesity.

## 1. Introduction 

Obesity is a risk factor for many chronic diseases, including diabetes mellitus, hypertension, dyslipidaemia, and ischemic heart disease [1, 2]. The association of obesity with these cardiovascular and metabolic comorbidities is complex, but the central pattern of fat distribution, a characteristic of patients with metabolic syndrome (MS), seems to play an important role [3]. 

As several studies have reported low serum 25-hydroxyvitamin D (25(OH)D) levels in obese patients, vitamin D deficiency is currently considered an obesity comorbidity [4–7]. However, it remains uncertain whether the presence of MS and insulin resistance contribute to the greater rates of hypovitaminosis D observed in obese individuals.

The present study was undertaken to assess the association of vitamin D status with the degree of obesity, the presence of MS and insulin resistance in overweight and obese subjects.

## 2. Materials and Methods 

A cross-sectional study was conducted on 343 Caucasian patients who were overweight or obese and who attended the obesity clinic during months of reduced sun exposure (from October to April). Exclusion criteria were conditions that could affect vitamin D concentrations, such as malabsorption, sunlight allergies, current treatments with calcium and vitamin D derivatives, treatment with antiepileptic drugs or rifampicin, kidney or liver insufficiency, calcium disturbances, and menopause. The study protocol was approved by the Hospital Ethics Committee, and all patients gave their informed consent. 

In the study visit we recorded anthropometric data (weight, height, body mass index (BMI), and waist circumference) and the presence or absence of MS. Waist measurements were taken using the upper border of the iliac crest as a reference, in accordance with NIH definitions [8]. Blood pressure was measured twice in seated subjects after a 5 min rest using a mercury sphygmomanometer. All analyses were performed after an overnight fast.

Based on their BMI, the patients were classified as being overweight (BMI 25–29.9 kg/m^2^) or having type I obesity (BMI 30–34.9 kg/m^2^), type II obesity (BMI 35–39.9 kg/m^2^), or type III obesity (>40 kg/m^2^). The diagnosis of MS was established according to the Adult Treatment Panel III (ATP III), which defines MS as the presence of three or more of the following criteria: abdominal obesity (waist circumference > 102 cm in men or >88 cm in women), triglycerides > 150 mg/dL (1.69 mmol/L), high density cholesterol < 40 mg/dL (1.04 mmol/L) in men or <50 mg/dL (1.30 mmol/L) in women, blood pressure > 130/85 mmHg and fasting glucose > 100 mg/dL (6.1 mmol/L) or previous diagnosis of diabetes [9]. 

Serum insulin concentrations were measured by an automated, solid-phase, two-site chemiluminescent immunometric assay (Immulite 2000, Diagnostic Products Corp., Los Angeles, CA), with 8% crossreactivity with proinsulin and a total analytical imprecision less than 7.5% for values between 55 and 2100 pmol/L (7.7 and 291 mIU/mL). The HOMA index was calculated as (glucose (mmol/L) *·* insulinemia (*μ*UI/mL))/22.5.

Serum 25(OH)D concentrations were measured using a commercial radioimmunoassay (Incstard Corp., Stillwater, MN, USA), with a normal range 25 to 150 nmol/L. 25(OH)D concentrations were defined as normal (>50 nmol/L), or hypovitaminosis D (<50 nmol/L) according to Lips criteria [10]. 

The data were analyzed using SPSS 17.0 statistical package (SPSS Inc.). Student's *t*-test and the Mann-Whitney *U* test were used to analyze independent samples, and the relationship between qualitative variables was assessed using a chi-square test. A multiple logistic regression analysis using a backward method was performed; hypovitaminosis D was the dependent variable and MS, obesity degree, age, and HOMA index (categorized into 3 groups according to quartiles 1 and 3) were the independent variables. Interactions of MS with obesity degree and HOMA were assessed.

## 3. Results

The clinical and biochemical characteristics of the 343 patients are shown in Table 1. The patients with MS were older (51 ± 9 versus 41 ± 11 years), and they had higher BMIs (39.4 ± 8.8 versus 34 ± 8 kg/m^2^), waist circumferences (116 ± 11 versus 105 ± 14 cm), and HOMA indexes (5.3 ± 2.9 versus 2.6 ± 1.8). They presented with lower 25(OH)D levels than the patients without MS (43.3 ± 29 versus 55.3 ± 30 nmol/L). Seventy-eight percent of subjects with MS and 49.2% of subjects without MS presented hypovitaminosis D (*P* < .000).

Fifty-two percent of the population studied presented hypovitaminosis D. The patients with hypovitaminosis D had higher BMIs (37.4 ± 9.6 versus 31.7 ± 5.3 kg/m^2^), waist circumferences (111 ± 15.4 versus 101 ± 12.1 cm), and HOMA indexes (3.32 ± 2.3 versus 2.6 ± 1.8) than the patients with normal 25(OH)D levels. The proportion of patients with the MS was higher in patients with hypovitaminosis D than in patients with normal vitamin D levels (19.8% versus 6.2%; *P* < .000). Distribution of vitamin D status according to the degree of obesity is shown in Table 2. In patients without MS, the prevalence of hypovitaminosis D exceeded the prevalence of normal vitamin D levels only when the BMI was >35 kg/m^2^, especially in those with a BMI > 40 kg/m^2^, while, in patients with MS, there was a predominance of hypovitaminosis D at all degrees of obesity (data not shown). When we excluded the patients with a BMI ≥ 40 kg/m^2^, the proportions of patients with normal vitamin D levels and hypovitaminosis D were 58% and 42%, respectively, in patients without MS and 36.8% and 73.2%, respectively, in patients with MS (*P* = .002). 

Finally, the multiple logistic regression analysis showed that both the degree of obesity (*P* = .001) and the presence of MS (*P* = .023) were independently associated with the presence of hypovitaminosis D. The odds ratio for hypovitaminosis D was 4.9 (confidence interval (CI), 2–14) for type III obesity versus overweight patients, while the odds ratio for hypovitaminosis D was 2.7 (confidence interval (CI), 1.14–6.4) for patients with MS versus patients without MS. HOMA index and interactions of MS with obesity and HOMA were not significant. Unadjusted and adjusted odds ratios of having hypovitaminosis D according to the presence of MS are shown in Table 3. 

## 4. Discussion

The main finding of our study was that the relationship between vitamin D and MS was independent of the degree of obesity, adding to our understanding of the relationship between vitamin D and MS in obese subjects.

The link between hypovitaminosis D and obesity has been widely demonstrated, particularly in the extremely obese patients [4–7]. Several hypotheses have been proposed to explain this association. It has been suggested that obese subjects have less exposure to sunlight, an inadequate intake of vitamin D, and decreased bioavailability of vitamin D due to enhanced uptake and clearance by adipose tissue [11, 12]. In our study, patients with hypovitaminosis D had higher BMIs, and the prevalence of hypovitaminosis D was increased in patients with higher degrees of obesity, especially those with a BMI > 40 kg/m^2^. Our data thus confirm the findings of previous studies where the inverse association between obesity and hypovitaminosis D was greater in extremely obese patients [13].

There are compelling data linking hypovitaminosis D with a higher prevalence of MS in a variety of populations. In the National Health and Nutrition Examination Survey (NHANES III) [14], mean 25(OH)D concentrations were lower in subjects with MS than in subjects without MS (67.1 versus 75.9 nmol/L), and in another recent study in adults in the US [15], the odds ratio of having MS in patients in the highest quintile for vitamin D levels compared with those in the lowest quintile was 0.26. Similar results have been found in European [16–19] and Asian [20, 21] populations. Studies conducted on children, adolescents, and postmenopausal women also confirm an association of vitamin D with MS, even after controlling for BMI [22–25]. Studies conducted on obese subjects, however, have shown conflicting results. Rueda et al. [26] and Hjelmesæth et al. [27] failed to show a significant association between vitamin D and metabolic syndrome in severely obese subjects, and McGill et al. [28] also reported no association between vitamin D concentrations and the prevalence of metabolic syndrome in overweight and obese subjects. By contrast, Botella-Carretero et al. [29] found that extremely obese subjects with MS had a higher prevalence of hypovitaminosis D than patients without MS (60.9 versus 33.3%, resp.) despite having similar BMIs. Moreover, Al-Daghri et al. [30] recently found that regular exposure to sunlight resulted in increased levels of vitamin D and a decreased prevalence of MS in subjects with overweight and obesity. In our study, the odds ratio for hypovitaminosis D was 2.7 for patients with MS versus patients without MS, irrespective of the degree of obesity. These findings provide support for an association of vitamin D deficiency with MS in overweight and obese subjects and suggest that this association is independent of obesity degree. 

Another factor that has been linked to vitamin D deficiency is insulin resistance, a key element in the development of MS [31]. Data from the Framingham Offspring Study show an inverse association of 25(OH)D with glycemia, insulin, and the HOMA index after adjustment for BMI [32]. In another prospective study assessing the relationship between calcidiol and hyperglycemia risk, the authors confirm an inverse association between calcidiol and the HOMA index, including after adjustment for BMI, age and seasonality [18]. In patients with MS, the results are contradictory. One recent report did not find an association between 25(OH)D and the HOMA index after adjusting for BMI [33], while in another study the association between vitamin D and MS was attenuated after adjustment for the HOMA index, suggesting that insulin resistance may partly account for the relation between vitamin D and MS [17]. Our work showed that patients with vitamin D deficiency had higher HOMA indexes than patients with normal vitamin D status. However, this association lost significance in the multiple logistic regression analysis, suggesting either the absence of an association between these two variables or a lack of power to detect an existing association.

The main strength of our work lies in patient selection, reinforcing the external validity of our findings. First, we selected those patients who attended the obesity clinic during months of reduced sunlight, when differences in sun exposure between individuals are negligible. Second, all of our patients were Caucasian, thus limiting the influences of ethnicity on vitamin D metabolism. Furthermore, we excluded the presence of renal or liver diseases, several drugs and malabsorption, which are all known to interfere with vitamin D metabolism [34–38], and we took into account that calcium, phosphorus, PTH, and estrogens may modulate vitamin D synthesis and alter final 25(OH)D concentrations [10, 39–41]. The main limitation of our study is that the low number of patients did not allow us to analyze the link between vitamin D and MS in each BMI group separately. Moreover, since we used a cross-sectional design, we cannot establish whether the relations between MS and hypovitaminosis D are causal.

In summary, our study confirms the relationship of vitamin D status with the degree of obesity and MS and suggests that the association between vitamin D and MS is independent of the degree of obesity. Further prospective studies are needed to assess causality and to determine the clinical significance of these findings.

## Figures and Tables

**Table 1:** Patient characteristics (*n* = 343).

Age (years)	42 ± 11
Men (%)	34.1
Weight (Kg)	92.7 ± 22.4
BMI (Kg/m^2^)	34.7 ± 8.3
25–29.9 (%)	34.0
30–34.9 (%)	28.9
35–39.9 (%)	15.2
>40 (%)	21.3
Waist (cm)	107 ± 15
Metabolic syndrome (%)	13.4
Glycemia (nmol/L)	5.2 ± 1.36
Insulinemia (uUI/mL)	12.6 ± 8.0
HOMA	2.9 ± 2.1
Calcidiol (nmol/L)	53.7 ± 29.8

Data presented as means and standard deviations and percentages of patients.

BMI: body mass index.

HOMA: homeostasis model assessment.

**Table 2:** Vitamin D status according to the degree of obesity.

Obesity degree	Vitamin D status	
	Normal	Hypovitaminosis D
Overweight	20.1% (*n* = 69)	14.6% (*n* = 50)
Type I obesity	16.6% (*n* = 57)	12.2% (*n* = 42)
Type II obesity	6.7% (*n* = 23)	8.5% (*n* = 29)
Type III obesity	3.5% (*n* = 12)	17.8% (*n* = 61)

Total	46.9 (*n* = 161)	53.1 (*n* = 182)

Data presented as percentages and number of patients. *P* = .000, according to chi-square test.

**Table 3:** Unadjusted and adjusted odds ratios (OR) of having hypovitaminosis D in the presence of MS.

	OR (CI 95%)	*P*
Model 1*	3.723 (1.782–7.777)	.000
Model 2**	2.817 (1.287–6.169)	.010
Model 3***	2.71 (1.147–6.401)	.023

*Unadjusted.

**Adjusted for degree of obesity and age.

***Adjusted for degree of obesity, age, and HOMA (homeostasis model assessment).

MS: metabolic syndrome.
